# Regulation of *UGT1A1 *and *HNF1 *transcription factor gene expression by DNA methylation in colon cancer cells

**DOI:** 10.1186/1471-2199-11-9

**Published:** 2010-01-22

**Authors:** Anne-Sophie Bélanger, Jelena Tojcic, Mario Harvey, Chantal Guillemette

**Affiliations:** 1 Pharmacogenomics Laboratory, Centre Hospitalier Universitaire de Québec (CHUQ), 2705 Laurier, Quebec, G1V 4G2, Canada; 2 Faculty of Pharmacy, Laval University, Quebec, G1V 0A6, Canada

## Abstract

**Background:**

UDP-glucuronosyltransferase 1A1 (UGT1A1) is a pivotal enzyme involved in metabolism of SN-38, the active metabolite of irinotecan commonly used to treat metastatic colorectal cancer. We previously demonstrated aberrant methylation of specific CpG dinucleotides in UGT1A1-negative cells, and revealed that methylation state of the *UGT1A1 *5'-flanking sequence is negatively correlated with gene transcription. Interestingly, one of these CpG dinucleotides (CpG -4) is found close to a HNF1 response element (HRE), known to be involved in activation of *UGT1A1 *gene expression, and within an upstream stimulating factor (USF) binding site.

**Results:**

Gel retardation assays revealed that methylation of CpG-4 directly affect the interaction of USF1/2 with its cognate sequence without altering the binding for HNF1-alpha. Luciferase assays sustained a role for USF1/2 and HNF1-alpha in *UGT1A1 *regulation in colon cancer cells. Based on the differential expression profiles of *HNF1A *gene in colon cell lines, we also assessed whether methylation affects its expression. In agreement with the presence of CpG islands in the *HNF1A *promoter, treatments of UGT1A1-negative HCT116 colon cancer cells with a DNA methyltransferase inhibitor restore *HNF1A *gene expression, as observed for *UGT1A1*.

**Conclusions:**

This study reveals that basal *UGT1A1 *expression in colon cells is positively regulated by HNF1-alpha and USF, and negatively regulated by DNA methylation. Besides, DNA methylation of *HNF1A *could also play an important role in regulating additional cellular drug metabolism and transporter pathways. This process may contribute to determine local inactivation of drugs such as the anticancer agent SN-38 by glucuronidation and define tumoral response.

## Background

Irinotecan (CPT-11) is a topoisomerase I inhibitor and one of the main cytotoxic agent for treatment of advanced metastatic colorectal cancer [1-3]. *In vivo*, irinotecan is converted to 7-ethyl-10-hydroxycamptothecin (SN-38), by a carboxylesterase-mediated hydrolysis, a metabolite ~1000-fold more active as topoisomerase I inhibitor than irinotecan [4,5]. The elimination pathway of the active metabolite SN-38 is primarily through glucuronidation, which is mainly mediated by the UDP-glucuronosyltransferase (UGT) 1A1 enzyme [6-8]. Low rates of SN-38 glucuronidation in tumor sites increase the level of the active compound that could lead to higher sensitivity to irinotecan. In contrast, high levels of UGT activity and expression were associated with an increase of SN-38 resistance in colon cancer cells [9]. Therefore, the regulation of *UGT1A *gene expression together with other mechanisms altering its protein activity should be considered in tumor resistance to SN-38.

Epigenetic regulation is a key mechanism to either activate or silence gene transcription, and abnormal epigenetic regulation has been described as an important characteristic of tumor malignancy and progression [10,11]. Furthermore, abnormal methylation of genes is a more common mechanism influencing gene activity than inheritable genetic mutations [12], and might confer intrinsic drug resistance to chemotherapeutic treatment. More specifically, colorectal cancer (CRC) is commonly associated with an abnormal methylation of CpG rich site (CpG island) in promoter region of multiple loci [10,11]. Indeed, a subset of CRC exhibit promoter methylation in multiple genes, referred to as the CpG island methylator phenotype [10,13]. Hence, it is rational to propose that abnormal epigenetic regulation of SN-38-metabolizing genes would be a drug resistance mechanism.

We previously demonstrated aberrant methylation of specific CpG-rich regions in UGT1A1-negative cells (HCT116, HCT-15, and COLO-320DM), and such events result in complete repression of *UGT1A1 *transcriptional activity [14]. DNA methylation may repress transcription by sterically hindering the binding of activating transcription factors (TFs) to their recognition sites [15-17]. Similarly, treatment with DNA methylation inhibitors enable binding of positive TFs and lead to gene reactivation [18]. In our previous report, treatment with demethylating and histone deacetylase inhibitor agents had the capacity to reverse aberrant hypermethylation and to restore *UGT1A1 *expression in hypermethylated UGT1A1-negative cells HCT116, but not in hypomethylated cells. Loss of *UGT1A1 *methylation was further associated with an increase in UGT1A1 protein levels and with an enhanced SN-38 inactivation, by 300% in HCT116 colon cancer cells [14]. In addition, human colon cancer cells has revealed that hypomethylation of the *UGT1A1 *5'-flanking sequence (-540 to -1) is important for *UGT1A1 *transcription. More specifically, the extent of *UGT1A1 *promoter methylation between CpG-1 (-4nt relative to the ATG) and -4 (-99nt relative to the ATG) of the promoter was shown to significantly predict *UGT1A1 *gene expression in colon cancer cell lines [14]. It is proposed that DNA methylation would alter the binding affinity of some important positive TFs.

In this report, we identified TF(s) that bind and influence transcriptional activity of *UGT1A1 *proximal promoter and determined whether methylation of CpG dinucleotides in this genomic region prevents binding of positive transcription factors.

## Results

### USF1/2 and HNF1-alpha bind the *UGT1A1 *gene promoter and activate transcription

**Figure 1:**
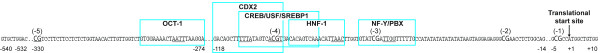
**Putative transcription factor binding sites in human *UGT1A1 *proximal promoter**. Schematic representation of the *UGT1A1 *gene sequence between positions -540 and +10 (relative to the transcription start site). Positions of CpG dinucleotides are shown. Sequences of the putative transcription factor binding sites are boxed. The recognition core sequence for each transcription factor is underlined.

By using a computer-based approach (MatInspector; http://www.genomatix.de/), several putative TF binding sites were observed in *UGT1A1 *5'-flanking sequence (-540 to -1), namely NF-Y (-57 to -73), HNF1-alpha (-79 to -95), CDX2 (-98 to -118), USF (-87 to -110) and OCT1 (-274 to -293) binding sites encompassing CpG-1 to -5 (Figure 1). Among those TFs, HNF1-alpha, CDX2 and OCT1 have previously been shown to interact with some UGT1A isoforms [19-25], but the interaction with *UGT1A1 *was only demonstrated for HNF1-alpha [26]. Interestingly, the CpG-4 is included in the USF recognition core sequence, the CpG-3 is part of the NF-Y/PBX binding site, and the HNF1 response element (HRE) is found between CpG-3 and -4.

**Figure 2:**
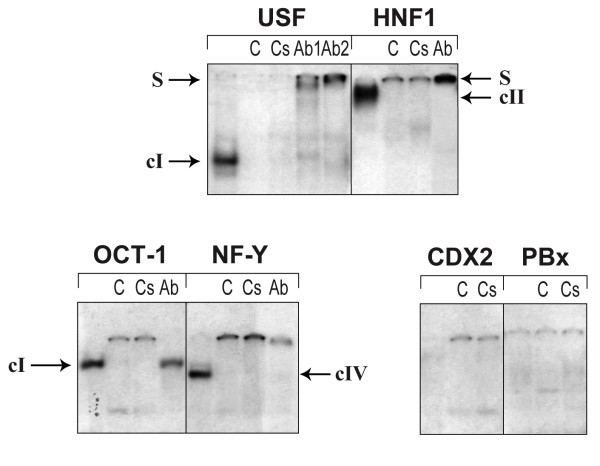
**Interaction of transcription factors with the *UGT1A1 *promoter using protein nuclear extracts of UGT1A1-expressing HT29 colon cancer cells**. EMSAs were performed with 32P-end-labeled probes. Nuclear protein extracts (5 μg) from HT29 cells were pre-incubated in the presence of either no competitor or 100-fold molar excess of cold competitor oligonucleotides (C) or consensus sequence (Cs). For supershift assays, 2 μg of the indicated antibody (Ab) were added directly after the addition of the labeled probe. For USF assays, Ab1 and Ab2 are directed against USF1 and USF2, respectively.

To address whether NF-Y, HNF1-alpha, CDX2, USF1/2, and OCT1 might specifically bind to the proximal promoter of *UGT1A1*, electrophoretic mobility shift assays (EMSA) were performed using nuclear protein extracts from HT29 cells, and synthetic double-stranded oligonucleotides derived from the *UGT1A1 *promoter sequence and containing each putative TF binding site (see Table 1). DNA-protein complexes are formed with all transcription factor-associated oligonucleotide probes, except for CDX2 and PBX (Figure 2). Each specific DNA-protein complex was competed by the addition of 100-fold molar excess of either the consensus recognition sequence or the unlabeled probe, thus providing evidence for specific binding. To further support the identity of DNA-binding proteins, we performed binding assays in presence of specific antibodies. The addition of mouse monoclonal anti-USF1 and anti-USF2 led to the formation of a supershifted DNA-protein complex (designated as S in figure 2) with the USF response element (URE)-containing probe, likely indicating that both USF1 and USF2 might bind to *UGT1A1 *promoter. Similar results were observed with the specific anti-HNF1-alpha and NF-Y antibodies, whereas the presence of anti-OCT1 antibody did not affect the formation of DNA-protein complex III with the OCT1-specific probe. The later likely indicates that an unknown DNA-binding protein might interact with this *UGT1A1 *promoter sequence.

**Table 1:** List of oligonucleotides

Transcient transfection experiement			
Transcription factor^a^			
OCT1	5'	GGTCTGTGGAAATACT**GGCC**TAATGGATCCTGAGGTTC	3'
CDX2	5'	GTGGACTGACAGCTTTT**CGCC**GTCACGTGACACAGTC	3'
USF	5'	GCTTTTTATAGTCA**AT**TGACACAGTCAAAC	3'
HNF1	5'	CACAGTCAAACAT**GAG**CTTGGTGTATCGA	3'
NF-Y	5'	CTTGGTGTATCGA**CCT**GTTTTTGCCATAT	3'
			
**Electrophoretic mobility shift assays**			

Putative TFs binding motif in UGT1A1 proximal promoter^a^			
OCT1	5'	GTGGAAATACT**AATT**TAATGGA	3'
CDX2	5'	GACTGACAGCTT**TTTA**TAGTCA	3'
CREB/USF/SREBP1	5'	TTTTTATAGTC**ACGTG**ACACAGTC	3'
HNF1	5'	CACAGTCAAACAT**TAAC**TTG	3'
NF-Y	5'	CTTGGTGTATCGA**TTGG**TTTTTGCCAT	3'
PBX	5'	TGTATC**GATT**GGTTTTTGCCATATAT	3'
			
Consensus oligonucleotide probes used in competition assays^a^			
OCT1 consensus oligo	5'	TGTCGAATGCA**AATC**ACTAGAA	3'
CDX2 consensus oligo	5'	GTGCAATAAAAC**TTTA**TGAGTA	3'
USF consensus oligo	5'	CACCCGGTCA**CGTG**GCCTACACC	3'
HNF1 consensus oligo	5'	CCAGGTTAATGAT**TAAC**CCA	3'
NF-Y consensus oligo	5'	AGACCGTACGTGA**TTGG**TTAATCTCTT	3'
PBX consensus oligo	5'	CGAATT**GATT**GATGCACTAATTGCAG	3'
			
Oligonucleotide probes of the *UGT1A1 *promoter with CpG sites^b^			
CpG-4/USF	5'	AGCTTTTTATAGTCA**CG**TGACACAGTCAAACAT	3'
CpG-4/USF-HNF1	5'	TCA**CG**TGACACAGTCAAACATTAACTTGGT	3'
			
**Reverse transcription (RT)-PCR**			

Target gene			
HNF-1*α *Forward	5'	GGTCATGAGCTTCGTCAACC	3'
HNF-1*α *Reverse	5'	GCAGGAAGAGATCCGATTCC	3'
USF-1 Forward	5'	GGCAGCTGAGACGGCCTTGG	3'
USF-1 Reverse	5'	TGGCCCCCATTCTCAGTTCGGA	3'
USF-2 Forward	5'	CTGCCTCTGTGCCCCCAGGT	3'
USF-2 Reverse	5'	TTGTCCCTCCGCCTCCGCTC	3'
GAPDH Forward	5'	TTGGTATCGTGGAAGGACTCA	3'
GAPDH Reverse	5'	TGTCATCATATTTGGCAGGTTT	3'

^a ^TF recognition core sequence is underlined.

^b ^*UGT1A1 *promoter CpG sites are underlined.

**Figure 3:**
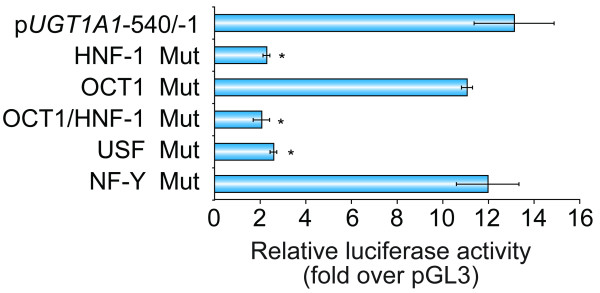
**Disruption of HNF1-alpha and USF recognition sequences decreases *UGT1A1 *basal expression in HT29 cells**. Mutational analysis of the putative HNF1, OCT1, USF and NF-Y binding sites in the *UGT1A1 *proximal promoter. Relative luciferase activity is expressed as a fold induction over the promoterless pGL3-basic vector (set to value = 1). *Columns*, mean of triplicates of three independent experiments; *bars*, SD; *, P < 0.05

To investigate the functional importance of these DNA-protein interactions, namely with USF1/2, HNF1-alpha, NF-Y, and OCT1, upon *UGT1A1 *proximal promoter activity, we disrupted either of their predicted recognition sequences in a 540 bp fragment of the human *UGT1A1 *gene promoter (p*UGT1A1*-540/-1). These constructions were introduced into the pGL3-luciferase reporter plasmid and transfected in UGT1A1-expressing HT29 cells. *UGT1A1 *proximal promoter activity was significantly attenuated by disruption of HRE and URE (Figure 3). In contrast, mutations in the NF-Y and OCT1 binding motifs had no effect on transcriptional activity. The result for OCT1 is in accordance with previous EMSA experiments. Accordingly, these results established that HRE and URE would play a role in positive regulation of the *UGT1A1 *gene expression.

### CpG methylation at the USF response element inhibits the formation of specific DNA-protein complex

**Figure 4:**
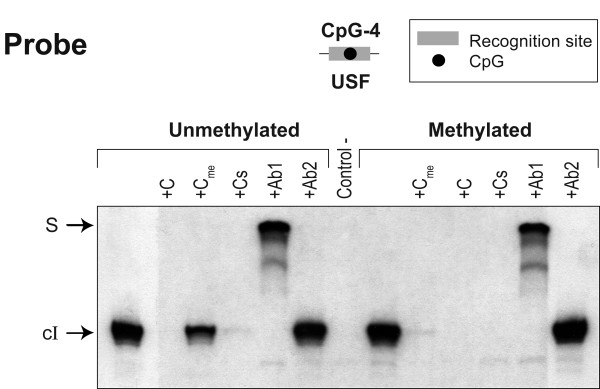
**Incubation with *in vitro *translated USF1 protein demonstrates that methylation of CpG-4 impedes USF1 binding**. EMSAs were performed with 32P-end-labeled probes. In vitro translated USF1 proteins were pre-incubated in the presence of either no competitor or 100-fold molar excess of cold competitor oligonucleotides (C) or consensus sequence (Cs). For supershift assay, 2 μg of the indicated antibody (Ab) were added directly after the addition of the labeled probe. Ab1 and Ab2 are directed against USF1 and USF2, respectively. In methylated probes, dC nucleotide is substituted by an internal methyl deoxyCytidine in CpG-4 dinucleotide.

As described above, the CpG-4 is part of the USF recognition core sequence. Therefore, we may expect that cytosine methylation at this site would hinder specific TF interaction. On the other hand, the HRE is found between CpG-3 and -4 dinucleotides and should intuitively not be affected by CpG-related DNA methylation. To investigate this, we firstly performed EMSAs using a double-stranded oligonucleotide probe including the URE. The oligonucleotide has been either methylated or not at the CpG-4 dinucleotide (Figure 4). Incubation of *in vitro *translated USF1 proteins with either methylated or unmethylated 32P-labeled probe resulted in the formation of specific DNA-protein complexes (identified as complex I). This indicates that 5-methylcytosine did not totally prevent the USF1 protein binding. However, the protein binding to unmethylated probe is less competed by 100-fold excess of methylated oligonucleotide than the unmethylated one, whereas binding to methylated probe is equally competed by either methylated or unmethylated cold oligonucleotide. It suggests that USF1 may interact with methylated DNA but have higher affinity for its unmethylated binding motif. The incubation of USF1 protein with either anti-USF1 or anti-USF2 antibody well demonstrated that USF1 specifically bind this *UGT1A1 *promoter sequence. The absence of supershifted complex with anti-USF2 antibody denotes that antibody-induced supershifted complex is not caused by a non-specific interaction with the antibody.

**Figure 5:**
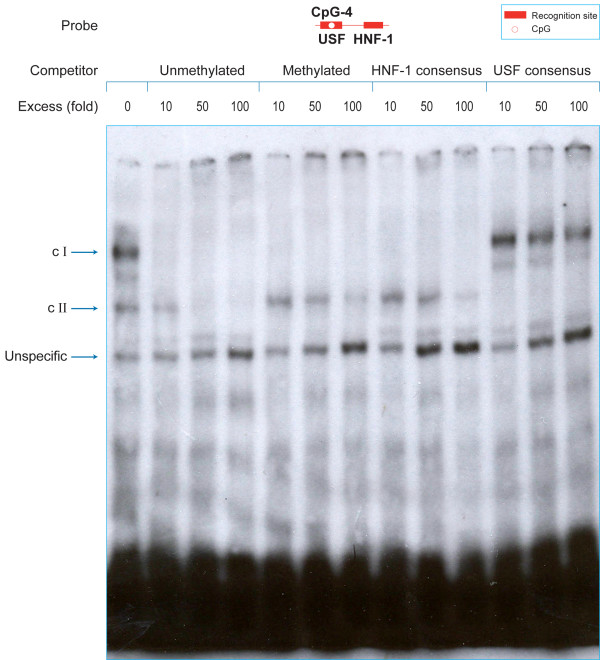
**Methylation of CpG-4 decreases USF1/2-associated complex stability**. EMSAs were performed with 32P-end-labeled probes. Nuclear protein extracts (5 μg) from HT29 cells were pre-incubated in the presence of either no competitor or 10-, 50-, 100-fold molar excess of cold competitors (unmethylated and methylated) or oligonucleotides including either a consensus binding site for HNF1-alpha or USF1/2. In methylated probes, dC nucleotide in CpG dinucleotides is substituted by an internal methyl deoxyCytidine.

In a second EMSA experiment, we examined the interaction between USF1/2 and HNF1-alpha (from HT29 nuclear protein extracts) with *UGT1A1 *promoter sequence including both TF binding sites. We used solely unmethylated oligonucleotide as probe, but either unmethylated or methylated oligonucleotide as cold competitor (Figure 5). We observed the formation of two specific and one unspecific DNA-protein complexes. By specific competition with HRE or URE-containing oligonucleotide, we determined that DNA-protein complex I and II are formed by HNF1-alpha and USF1/2, respectively. We noted that solely the specific complex I is equally competed by excess of both methylated and unmethylated cold competitor. In contrast the complex II, formed by USF1/2, is more competed by molar excess of unmethylated cold competitor. The unspecific DNA-protein complex is not competed by either competitor. The positive correlation between the unspecific complex intensity and the increase amount of competitor indicates a rise in probe availability for nonspecific DNA-binding proteins. In summary, this experiment further demonstrated that CpG methylation impairs DNA-binding for USF1/2 but not for HNF1-alpha, likely because its recognition site remains unaffected by CpG methylation in our experiment context.

### Upregulation of *HNF1A *gene expression is observed following treatment with the 5-Aza-dC demethylating agent in UGT1A1-negative cells

**Figure 6:**
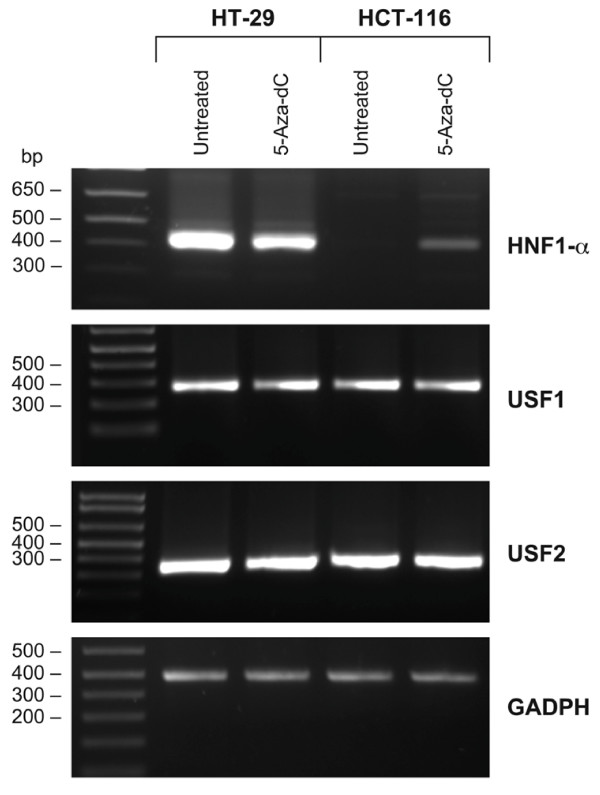
**Upregulation of *HNF1A *expression is observed following treatment with the 5-Aza-dC demethylating agent in UGT1A1-negative HCT116 colon cancer cells**. Rescue of *HNF1A *gene expression by 5-Aza-dC treatment of colon carcinoma cell lines. Total RNA from HT29 and HCT116 cells were randomly converted to cDNA and specific amplification for USF1, USF2, and HNF1 mRNAs were carried out. Expression of *USF1 *and *USF2 *mRNAs are not influenced by 5-Aza-dC treatment. *GAPDH *gene is used as internal control. Untreated; cells not exposed to 5-Aza-dC.

We presented that CpG methylation might affect *UGT1A1 *gene expression through alteration of cis-acting elements. However, evidence supports that DNA methylation-induced gene silencing is also caused by inhibition of trans-acting factor gene expression. We previously demonstrated that the UGT1A1-negative cell line HCT116 is able to express *UGT1A1 *following 5-aza-dC treatment [14]. We showed that such a gene induction is due, at least in part, by the demethylation of *UGT1A1 *promoter. Considering the importance of HNF1-alpha and USF1/2 in *UGT1A1 *gene expression and also that HCT116 cell line is known to be HNF1-negative, we sought to determine whether the *USF1 *and *USF2 *gene expression is influenced by the cell methylation status and whether *HNF1A *gene expression is restored in 5-aza-dC-treated HCT116 hypermethylated cells. As expected, the presence of *HNF1A *mRNA was undetectable by reverse PCR in untreated HCT116 cells. However, the 5-Aza-dC treatment induced the *HNF1A *gene expression (Figure 6). These data support that *HNF1A *is also modulated in these cells by methylation, as observed for *UGT1A1*. In fact, three regions in *HNF1A *-5kb promoter were predicted as CpG islands by the CpGPlot program (Emboss) (data not shown). On the other hand, we did not get any observable variation in both USF1 and USF2 gene expression following treatment with 5-aza-dC in both cell lines.

## Discussion

In this report, we predicted several putative TF binding sites in *UGT1A1 *proximal promoter using a bioinformatic tool and demonstrated by EMSA that HNF1-alpha, USF1/2, and NF-Y would bind to *UGT1A1 *proximal promoter. The influence of these TFs upon *UGT1A1 *transcriptional activity was then demonstrated by transient transfection in colon adenocarcinoma cell line HT29, and solely HNF1-alpha and USF1/2 have been shown to have significant impact.

Mutations in the HNF1-alpha motif resulted in a substantial reduction of *UGT1A1 *promoter activity in HT29 cells. This HRE located in the human *UGT1A1 *promoter consists of a very well conserved half-site and a more divergent one with respect to the consensus sequence, which is very similar to the one from mouse *UGT1A1 *promoter and rat albumin promoter [26,27]. Consistent with previous studies in mice, HNF1-alpha proteins failed to bind (data not shown) and trans-activate the human *UGT1A1 *promoter when the conserved half-site is altered (Table 1). Besides, our data indicate that the one half of the HRE palindromic sequence is sufficient for its recognition of the *UGT1A1 *promoter, and that HNF1-alpha is critical for *UGT1A1 *expression.

HNF1-alpha is well-known to be involved in regulation of several UGTs, including human *UGT2B7*, *UGT2B17*, *UGT1A1*, *UGT1A3*, *UGT1A4*, *UGT1A8*, *UGT1A9*, *UGT1A10 *and rat *UGT1A7 *[20,21,26,28-30]. Although the role of HNF1-alpha in the regulation of *UGT1A1 *has already been studied [26], the data were limited to transient transfections of the -617/+15 *UGT1A1 *promoter and its HNF1-deleted construct into UGT1A1-negative HEK293 kidney cells. In here, we emphasized the importance of HNF1-alpha in the regulation of *UGT1A1*, and in contrast to previous observations, data were demonstrated in cells with a known glucuronidation capacity.

As observed for HNF1-alpha, mutations in URE also resulted in a drastic reduction of the promoter activity in HT29 cells, supporting for the first time, a role for this TF in the regulation of *UGT1A1 *promoter. Upstream stimulatory factors, USF1 and 2 are late TFs able to interact as homo- and/or heterodimers on E boxes of CACGTG sequence [31-34]. USFs are ubiquitously expressed proteins that have been described as positive or negative regulators of numerous genes, including cyclin-cdk encoding genes, tumour suppressor genes, and growth factor networks [35,36]. To our knowledge, no interaction of USF1 or USF2 with phase II enzymes such as the UGT family members has been documented thus far.

While EMSA indicated that NF-Y might also bind *UGT1A1 *promoter, mutations in its binding motif did not significantly reduce the luciferase activity compared with the wild-type construct in HT29 cells, suggesting that basal promoter activity does not require direct interaction of this TF. Although informative, promoter-reporter constructs inadequately mimic the chromosomal context. It is now appreciated that chromatin-associated factors are various key determinants for specific gene expression [37]. Accordingly, we may not rule out that NF-Y would contribute to *UGT1A1 *gene expression in native cells.

The observation that URE includes a CpG dinucleotide contact point, which is critical for recognition by the USF proteins, prompted us to hypothesize that a USF E-box element that contained 5-methylcytosine in the CACGTG core might affect the binding for USF1/2. EMSA using unmethylated probe resulted in the formation of an USF-*UGT1A1 *complex. When methylated, URE-containing oligonucleotide competed poorly for USF1/2 binding, showing that specific methylation of CpG-4 dinucleotide decrease the affinity for USF1/2. It was previously shown that methylation at the CpG site, centrally located in the E-box motif (CACpGTG), strongly inhibits formation of DNA-protein complex [38,39] and negatively regulates gene expression. Single nucleotide polymorphisms, within the E-box core motif, also modulate gene regulation. Notably, a single G/C base transition within the USF E-box consensus of the thymidylate synthase gene, implicated in folate metabolism, prevents USF proteins from binding to their cognate sequence [40].

As we observed previously for *UGT1A1 *[14], data indicate that DNA methylation is one mechanism likely involved in the down-regulation of *HNF1A *gene expression in colon cells. DNA methyltransferase inhibitor treatment of UGT1A1-negative HCT116 colon cells restored *HNF1A *gene expression. 5-aza-dC-induced gene reactivation has two distinct requirements: 1) the reversal of promoter DNA hypermethylation, and 2) the presence of transcriptional activators competent for activation of the target promoter. Considering that HNF1-alpha is essential for *UGT1A1 *gene expression, the methylation of *HNF1A *gene promoter represents a second level of DNA methylation-mediated regulation, which highlights the complexity of epigenetic gene regulation. The modulation of *HNF1A *expression might also have impact on the regulation of other genes, notably on additional phase II enzymes including other UGTs [20,21,26,28-30], glutathione transferase [41], and sulfotransferase [42].

Interestingly, the UGT1A1-associated HRE, which is free of CpG dinucleotide, is located between CpG-3 and -4, and we demonstrated that methylation of proximal CpG dinucleotides is not sufficient to significantly alter HNF1-alpha binding *in vitro*. However, we may not rule out the importance of DNA methylation in the binding of HNF1-alpha *in vivo*, because such a DNA modification induces a repressive chromatin structure, and might restrain the accessibility of HNF1-alpha to its recognition sequence in *UGT1A1 *promoter. However, we suggest that *UGT1A1 *proximal promoter methylation may directly affect transcriptional activity by suppressing the interaction of USF1/2 with its cognate sequence.

Taken together, our results reveal that both HNF1-alpha and USF1/2 could play an important role in activating the transcription from *UGT1A1 *promoter. The interplay between HNF1-alpha and USF1/2 has been previously shown to be implicated in the liver-specific expression of the pyruvate kinase gene, in the regulation of three human class I alcohol dehydrogenase genes and in the constitutive expression of *CYP1A2 *[43-45]. Considering that UGT1A1-mediated glucuronidation is the primary route of irinotecan inactivation, it was suggested that the level of *UGT1A1 *expression might contribute to the differential chemosensitivity of colon tumors [46-48]. In a previous report, we showed that methylation of *UGT1A1 *promoter may conduct to reduction of gene expression level, leading to a lower UGT1A1 glucuronidation activity. Accordingly, positive *UGT1A1 *methylation in tumors, and subsequent repression of UGT1A1-associated metabolic pathways would be involved in retention of active SN-38 within colon cancer cells. This could lead to higher sensitivity to irinotecan. In contrast, the presence of high levels of UGT activity and expression was identified as a characteristic associated with a resistance phenotype to SN-38 in colon cancer cells, as supported by a previous report [9].

## Conclusions

This study reveals that basal *UGT1A1 *expression in colon cells is positively regulated by sequence-specific binding of HNF1-alpha and USF1/2, and negatively regulated by DNA methylation of CpG-4 located in the proximal *UGT1A1 *promoter. This suggests that CpG-4 methylation status might be a relevant indicator of *UGT1A1 *proximal promoter methylation and by consequence a potential epigenetic marker of *UGT1A1 *gene expression. Besides, epigenetic regulation of *HNF1A *gene could also play an important role in regulating additional cellular drug metabolism and transporter pathways. Altogether, the epigenetic regulation of *HNF1A *and *UGT1A1 *genes may contribute to determine local inactivation of drugs, such as the anticancer agent SN-38 by glucuronidation and define tumoral response to irinotecan. Further studies are required to examine this hypothesis.

## Methods

### Cell culture

Colon cancer cells HT29 and HCT116 were obtained from American Type Culture Collection (Manassas, VA). Cells were growth in the medium recommended by American Type Culture Collection. HT29 and HCT116 cells were kept in McCoy's 5A medium with 1.0 mmol/L sodium pyruvate (Sigma, Oakville, Ontario, Canada) added with 100 IU/mL penicillin, 50 μg/mL streptomycin, and 10% fetal bovine serum. Cells were incubated at 37°C in a humidified incubator with 5% CO_2_.

### Transient transfection

The preparation of *UGT1A1 *promoter-luciferase reporter constructs has been previously described [14]. For transient transfections, cells were plated into 24-well microplates at approximately 80 percent of confluence (150,000 Cells/well) in growth medium and transfected using Lipofectamine 2000 (Invitrogen, Carlsbad, CA) according to the manufacturer's instructions. *UGT1A1 *promoter-luciferase reporter constructs were used at 750 ng of plasmid per well and co-transfected with 5 ηg of Renilla luciferase plasmid. Cells were harvested 48 hours after transfection and assayed for promoter activity using the Dual-Luciferase Reporter Assay System. Luciferase activity was measured by using 40 μL of cell lysate in a 96-well plate on an LB96V microplate luminometer.

### Site-directed mutagenesis

Site-directed mutants were constructed with the The QuickChange mutagenesis kit (Stratagene) and the mutated oligonucleotides (Table 1). Mutations were confirmed by DNA sequencing before subcloning into the pGL3-Basic vector.

### Expression analysis by reverse transcription (RT)-PCR

Treatments with 5-Aza-dC were done as described previously [14] and RNA from HCT116 cancer cell line was extracted with Tri-Reagent (Molecular Research Center, Inc., Cincinnati, OH), as described in the manufacturer's protocol. RNA (1 μg) was converted to cDNA with SuperScript II RNase H-negative (Invitrogen, Burlington, Ontario, Canada) using the manufacturer's protocol in a 20 ul reaction volume. The amplification reactions were carried out in a 25 ul reaction volume including 1 ul of cDNA reaction, 10 pmol of each primer (listed in table 1), 200 pmol of dNTP, 1× Taq PCR buffer, 5% Acetamide, 0,5 unit of Taq DNA polymerase. 35 amplification cycles were peformed as follow: 20 sec at 95°C, 20 sec at 61°C, and 30 sec at 72°C. A 10 μL aliquot of each reaction mixture was electrophoresed on a 1% agarose gel containing ethidium bromide. The *GAPDH *gene was amplified as internal control.

### Preparation of nuclear extracts and *in vitro *production of USF protein

Nuclear extracts were prepared from trypsinized cells, centrifuged for 5 min at 800 × g and then resuspended in 5 ml of HB buffer (15 mM Tris-HCl (pH 8.0), 15 mM NaCl, 60 mM KCl, 0,5 mM EDTA), centrifuged at 800 × g for 5 min, resuspended in 100 μl of HB buffer supplemented with 0.05% Triton X-100 (Sigma), and centrifuged for 10 min at 1,000 × g, and the supernatant was discarded. The pellet was washed with 5 ml of HB buffer containing 0.05% Triton X-100 and 5 ml of HB buffer. Nuclei were incubated at 4°C for 30 min in 50 μl of HB buffer containing 360 mM KCl and centrifuged for 5 min at 10,000 × g, and the supernatant corresponding to the nuclear extract was collected. The concentration of protein in the extracts was determined using the Bradford method according to the manufacturer's recommendations. Human USF1 cDNA clone was kindly provided by Dr. Roger G. Roeder and described previously [49]. USF1 protein was synthesized *in vitro *using the TNT Quick Coupled Transcription/Translation System (Promega, Madison, Wisconsin, USA).

### Electrophoretic mobility shift assay (EMSA)

Protein-DNA interactions were carried out in a 20 ul reaction mixture including either 5 μg crude nuclear protein extract in (figure 2 and 5) or *in vitro *synthesized USF proteins (in figure 4), 50 mM HEPES (pH 7.8), 300 mM KCl, 1% Igepal, 30% glycerol, 1 mM DTT, 0.01 μg of poly(dI-dC), 0.05 μg of ssDNA, and 20 000 CPM of 32P-radiolabeled oligonucleotide. After incubation for 20 minutes at room temperature, the protein-DNA complexes were resolved onto 6% non-denaturing polyacrylamide gel electrophoresis at 4°C (when using nuclear extracts) and at room temperature (when using *in vitro *translated USF proteins). For competition experiments, unlabeled oligonucleotides were added in the reaction mixture at a molar excess of 100-fold (Figures 2 and 4), or 10 to 100-fold (figure 5). The oligonucleotides including consensus recognition sequence for transcription factors (Table 1) are derived from Transcruz gel shift oligonucleotides (SantaCruz Biotechnology, CA), except for CDX2[50] and HNF1[51]. For supershift assays, 2 μg of a monoclonal (Biogenex, San Ramon, California, USA) antibody to human CDX2 or polyclonal antibody to HNF1-alpha (C-19), NF-Y (CBF-B, C-18) OCT1 (C-21), USF1 (C-20) and USF2 (C-20) (Santa Cruz Biotechnology, Inc., Santa Cruz, California, USA) were added directly after the addition of labeled probe, with the exception of pre-incubation experiments where antibodies were pre-incubated for 30 min prior to probe addition.

## Authors' contributions

ASB: acquisition of data; analysis and interpretation of data; drafting of the manuscript. JT: acquisition of data; analysis and interpretation of data. MH: analysis and interpretation of data; critical revision of the manuscript for important intellectual content. CG: study concept and design; study supervision, critical revision of the manuscript for important intellectual content; obtained funding. All authors have read and approved the final version of the manuscript.
